# Editorial: Challenges in peripheral T-cell lymphomas: from biological advances to clinical applicability

**DOI:** 10.3389/fonc.2023.1325170

**Published:** 2023-12-13

**Authors:** Luís Alberto de Pádua Covas Lage, Juliana Pereira, Ryan A. Wilcox

**Affiliations:** ^1^ Department of Hematology, Hemotherapy & Cell Therapy, Faculty of Medicine – University of São Paulo (FM-USP), São Paulo, Brazil; ^2^ Laboratory of Medical Investigation in Pathogenesis and Directed Therapy in Onco-Immuno-Hematology (LIM-31), Faculty of Medicine – University of São Paulo (FM-USP), São Paulo, Brazil; ^3^ Hospital Alemão Oswaldo Cruz (HAOC), Department of Hematology and Oncology, São Paulo, Brazil; ^4^ Department of Internal Medicine, Division of Hematology and Oncology, University of Michigan, Ann Arbor, MI, United States

**Keywords:** peripheral T-cell lymphoma, pathogenesis, molecular biomarkers, epigenetic regulation, treatment

Peripheral (mature) T-cell lymphomas (PTCL) are a heterogeneous group of rare lymphoid malignancies derived from monoclonal proliferation of CD4+ T-helper cell subsets, CD8+ cytotoxic T-cells or natural-killer cells (NK). Although less prevalent than B-cell non-Hodgkin’s lymphomas (NHLs), understanding of their molecular pathogenesis and classification has significantly improved over the last decade. However, these advances have not always translated into therapeutic improvement, and thus treatment of the NK/T-cell lymphomas remain an area of unmet medical need. Therefore, most PTCL patients still have poor clinical outcomes and substantially shortened survival when compared to individuals with aggressive B-cell lymphomas. This Special Edition includes 15 scientific articles that compile the main biological, pathological and therapeutic advances recently obtained in this field of knowledge, aiming to improve the clinical outcomes of PTCL patients.

The classification, risk-stratification and treatment of most PTCL subtypes remain, in the words of those who contributed to this Research Topic, “a challenge” [Murga-Zamalloa and Inamdar; Zain and Kallam]. In this sense, Murga-Zamalloa and Inamdar reviewed the most recent updates implemented by the latest version of the World Health Organization’s classification of hematolymphoid tumors (WHO-HAEM 5th.), focusing on the most relevant diagnostic findings of PTCL, centered on histopathological basis and the description of new molecular markers. The discovery of new molecular biomarkers has contributed significantly to the elucidation of pathogenic mechanisms, prognostic stratification and implementation of therapeutic measures adapted to biological risk and directed against classically deregulated signaling pathways in the setting of different subtypes of PTCL. Thus, Zain and Kallam and Drieux et al. prepared two comprehensive reviews in this Research Topic describing the main recurrent mutations, changes in the gene expression profile (GEP) and deregulated intracellular signaling pathways in PTCL. Additionally, the authors clarify how such molecular advances influence the therapeutic management of individuals with different subtypes of PTCL, highlighting the promising role of new drug classes directed against key molecular targets in these neoplasms, such as epigenetic modifying agents (histone deacetylase inhibitors [HDAi] and DNA methyl transferase antagonists [hypomethylants, such as 5-azacytidine and decitabine]), therapies that target kinases (PI3K, JAK, SYK, ALK and Aurora A kinase inhibitors), immunotherapeutic agents (anti-CD30, anti-CCR4, anti-CD25), and drugs that modulate the tumor microenvironment (TME), such as lenalidomide and immune checkpoint inhibitors.

Notwithstanding well-described geographic differences in PTCL prevalence, largely attributed to differences in the epidemiology of virally-associated, particularly EBV-related [Drieux et al., Barros et al.] NK/T-cell lymphoma subtypes [Costa et al.], the most common PTCL subtype remains “not otherwise specified” [Weiss et al.]. While Weiss et al. described recent advances in our understanding of transcriptionally, genetically, and clinically distinct PTCL, NOS subsets, these advances are discussed in broader histopathologic [Murga-Zamalloa and Inamdar] and clinical [Zain and Kallam] contexts by other notable contributions to this Research Topic. The reviews conducted by Weiss et al., Zain and Kallam and Drieux et al. focuses on the central contribution of GEP to mitigate the diagnostic and prognostic heterogeneity of PTCL, NOS. Therefore, these authors highlight the categorization of two genetic subgroups within this category of PTCL, the TBX21 subtype, associated with Th1/NF-kB deregulation, and the subgroup with overexpression of the Th2/GATA3 transcription factor, related to deregulation of the PI3K/mTOR axis. Such articles reinforce the prognostic differences between both groups of PTCL, NOS and, highlighting the highly ominous prognosis of the GATA3 subgroup, as well as the potential therapeutic application of immunomodulatory agents in the TBX21 group and PI3K/mTOR inhibitors in the GATA3 subtype.

While the molecular advances have not yet led to a formal update in the latest WHO classification [Murga-Zamalloa and Inamdar], improved understanding in the molecular pathogenesis of angioimmunoblastic T-cell lymphomas (AITL), including the role of highly recurrent loss-of-function mutations in TET2 [Carty et al.], have led to significant changes in the classification of these and other highly related T follicular helper (TFH)-derived PTCL [Lage et al.; Marques-Piubelli et al.], and improved therapeutic strategies, with the advent of novel agents targeting the epigenome [Zain and Kallam; Drieux et al.; Carty et al.; Lage et al.; Marques-Piubelli et al.] or the TME [Drieux et al.; Lage et al.]. In this sense, Lage et al. and Marques-Piubelli et al. conducted two extensive reviews summarizing the main clinical-laboratory, pathogenic and histopathological aspects of nodal peripheral T-cell lymphomas with TFH-phenotype (nPTCL-TFH), focusing on AITL. In their article, Lage et al. highlight the main findings that make up the so-called “*immunodysplastic syndrome*”, characteristic of AITL, marked by different inflammatory and autoimmune features, as well as summarize the contribution of molecular changes involving the epigenetic machinery (IDH2, TET2, and DNMT3A) in the genesis of AITL [8]. Similarly, Marques-Piubelli et al. characterizes in their article the pathological findings of the main 3 subtypes of nPTCL-TFH in light of recent updates proposed by the WHO-HAEM5th. classification, list the antigenic expression profile of TFH-cells, and describe the main genomic-molecular findings of these malignancies. At the same time, in their review, Carty et al. dissects the biological role of TET2, particularly in nPTCL-TFH biology, highlighting its lymphomagenesis-promoting mechanisms and the potential of epigenetic therapies to improve the clinical outcomes of these neoplasms. Still in the therapeutic field of nodal PTCL, in the relapsed/refractory (R/R) setting, traditional chemotherapeutic agents do not provide an apparent advantage over novel drugs, and hence clinical trial participation is encouraged. However, Fante et al. highlight in their retrospective and single-center experience, the role of the all-oral and palliative regimen TEPIP (trophosphamide, etoposide, procarbazine, idarubicin and prednisolone) in elderly and frail patients with PTCL. In this study, the TEPIP regimen demonstrated competitive efficacy with a tolerable safety profile in a population with difficult-to-treat PTCL, making it a relatively safe and effective alternative in the palliative context of individuals with nodal PTCL.

As noted by Wu and Lim, the genetic landscape is a significant determinant of disease natural history and the response to therapy among anaplastic large cell lymphomas (ALCL). For example, those harboring recurrent *DUSP22* rearrangements are highly curable with the current standard of care based on BV-CHP (brentuximab-vedotin, cyclophosphamide, doxorubicin, and prednisone) regimen, whereas those harboring mutually exclusive *TP63* rearrangements are associated with dismal outcomes. While the molecular and genetic landscape has predictive value in PTCL, NOS [Weiss et al.] and ALCL [Wu and Lim], the natural history associated with AITL and other TFH-derived PTCL is notoriously variable, yet poorly understood. Hu et al. provide data suggesting that metabolic activity (total lesion glycolysis - TLG) by PET-CT may have prognostic implications in these patients, while Chen et al. provide preliminary evidence suggesting that this variable natural history may be explained, at least in part, by differences in tumor immune surveillance within the TME. In their pioneering study, Hu et al. identified that TLG was a strong predictor associated with poor overall survival in a cohort of 40 Chinese patients diagnosed with AITL and followed for more than 30 months. Additionally, these authors developed a new prognostic scoring system including different clinical-laboratory (IPI) and imaging/metabolic variables (TMTV, TLG and SUV max) in patients with AITL. By this score, three different risk-categories were identified, with 3-year overall survival (OS) estimates of 100%, 43%, and 25%, respectively. Interestingly, Chen et al. also conducted an unprecedented translational study, comparing clinicopathological findings, lymphocyte composition of the immune TME (TIL – tumor infiltrating lymphocytes), and gene expression profile between different subsets of AITL. In this study, the authors demonstrated that CD8-predominant AITL presents a very peculiar immune pattern, characterized by compromised anti-tumor immunity, immunosuppressive microenvironment, more severe clinical manifestations and worse survival, thus clarifying the heterogeneity of this clinicopathological entity.

The TME plays a central role in both the suppression of host anti-tumor immunity, but its constituents also directly promote T-cell lymphomagenesis by providing ligands for corresponding antigen, costimulatory, and cytokine receptors that are variously expressed across the PTCL spectrum. Not surprisingly then, the TME´s role in T-cell lymphomas generally, including CTCL [Miyashiro and Sanches], is a recurring theme across many papers included in this Research Topic, and has significant therapeutic implications in the era of checkpoint blockade [Costa et al.; Weiss et al.] and novel cellular therapies [Couto et al.]. Concerning to this last Research Topic, in their review article, Couto et al. discusses the use of autologous hematopoietic stem cell transplantation (ASCT) and allogeneic transplantation (allo-SCT) as therapeutic strategies for PTCL, as well as approaches based on the advancement of cellular therapy techniques, in addition to their limitations in the PTCL scenario, proposing some approaches to overcome them.

While the papers included in this Research Topic are diverse, a unifying theme is abundantly clear - improved understanding of the transcriptional, genetic, and TME-dependent drivers across the spectrum of NK/T-cell neoplasms has led to improved disease classification and risk-stratification, but has also expanded the menu of novel agents available on the therapeutic smorgasbord, and thus provides hope for a brighter future for new patients afflicted with these aggressive neoplasms.

## Author contributions

LL: Writing – review & editing. JP: Writing – review & editing. RW: Writing – review & editing.

